# Bones and genes: resolution problems in three Vietnamese species of *Crocidura* (Mammalia, Soricomorpha, Soricidae) and the description of an additional new species

**DOI:** 10.3897/zookeys.313.4823

**Published:** 2013-07-02

**Authors:** Paulina D. Jenkins, Alexei V. Abramov, Anna A. Bannikova, Viatcheslav V. Rozhnov

**Affiliations:** 1 The Natural History Museum, Cromwell Road, London SW7 5BD, UK; 2 Zoological Institute, Russian Academy of Sciences, Universitetskaya nab., 1, Saint-Petersburg 199034, Russia; 3 Lomonosov Moscow State University, Vorobievy Gory, Moscow 119992, Russia; 4 A.N. Severtsov Institute of Ecology and Evolution, Russian Academy of Sciences, Leninskii pr., 33, Moscow 119071, Russia; 5 Joint Vietnam-Russian Tropical Research and Technological Centre, Nguyen Van Huyen, Nghia Do, Cau Giay, Hanoi, Vietnam

**Keywords:** *Crocidura*, new species, morphology, molecular analysis, geographical variation

## Abstract

Recent investigations of Southeast Asian white toothed shrews belonging to the genus *Crocidura* have revealed discrepancies between the results of morphological and molecular studies. The following study concerns three species of *Crocidura* occurring in Vietnam, namely *Crocidura attenuata*, *Crocidura tanakae* and *Crocidura wuchihensis*, and an undescribed fourth species revealed by molecular analysis. For many years *Crocidura attenuata* has been known to occur in Vietnam but, until very recently, the morphologically similar and comparably sized *Crocidura tanakae* was believed to be restricted to Taiwan. Following several molecular studies over the last few years, this species is now believed to be considerably more widespread and recognised as occuring also in Vietnam. The results of one of these recent molecular studies also revealed the presence of an undescribed species of *Crocidura*, similar in size and morphology to *Crocidura wuchihensis*, which is herein described. Data are provided on geographical variation in Vietnam and the problems of defining morphologically similar yet molecularly disparate species are discussed.

## Introduction

From the late 1990s there have been several intensive surveys of the small mammal fauna in various localities in Vietnam, resulting in the discovery of a number of species new to science. Before that time only three species of *Crocidura* had been recorded from Vietnam: *Crocidura attenuata* Milne Edwards, 1872, *Crocidura fuliginosa* (Blyth, 1855) and *Crocidura indochinensis* Robinson & Kloss, 1922 (Van Peenen et al. 1969, Heaney and Timm 1983). Lunde et al. (2003) recorded the occurrence of a fourth species, *Crocidura wuchihensis* Shaw, Wang, Lu & Chang 1966 in northern Vietnam. This was followed by a spate of descriptions of new species of *Crocidura* based entirely on morphology: *Crocidura kegoensis* Lunde, Musser & Ziegler, 2004; *Crocidura sokolovi* Jenkins, Abramov, Rozhnov & Makarova, 2007; *Crocidura zaitsevi* Jenkins, Abramov, Rozhnov & Makarova, 2007; *Crocidura annamitensis* Jenkins, Lunde & Moncrieff, 2009; *Crocidura guy* Jenkins, Lunde & Moncrieff, 2009; *Crocidura phuquocensis* Abramov, Jenkins, Rozhnov & Kalinin, 2008a; *Crocidura phanluongi* Jenkins, Abramov, Rozhnov & Olsson, 2010.

Molecular studies were also being carried out during this period. Significant studies included those of Ohdachi et al. (2006) investigating the mitochondrial cytochrome *b* gene sequences of Soricidae; Esselstyn et al. (2009), Esselstyn and Brown (2009) and Esselstyn and Oliveros (2010) studying mitochondrial and nuclear genes of *Crocidura*. These were broad based studies covering wide geographical regions of Southeast Asia, Indonesia and the Philippines but some samples of Vietnamese *Crocidura* were included in their analyses. Bannikova et al. (2011) studied two mitochondrial genes, cytochrome *b* (cyt *b*) and cytochrome *c* oxidase subunit I gene (COI), of Vietnamese *Crocidura* collected at various localities ranging from the north to the south of the country.

While the molecular studies of Vietnamese material confirmed some of the results of the contemporaneous morphological studies, a number of anomalies were equally revealed, indicating the presence of several morphologically similar but molecularly distinct taxa. Investigation of these incongruent results is the subject of this current study.

## Background to identification of species based on DNA analysis

### *Crocidura attenuata* and *Crocidura tanakae*

*Crocidura attenuata* Milne Edwards, 1872 described originally from Szechuan, China, was regarded as a widespread and common species known throughout much of Asia, including many localities from northern to southern Vietnam.

*Crocidura tanakae* Kuroda, 1938 from Taiwan was originally described as a full species but was subsequently considered to be either a synonym or subspecies of *Crocidura attenuata* (Ellerman and Morrison-Scott 1951, Jameson and Jones 1977, Fang et al. 1997, Jiang and Hoffmann 2001, Han et al. 2002). Motokawa et al. (2001) demonstrated that the karyotype of Taiwanese specimens differed from that of *Crocidura attenuata* from mainland southern China and suggested that it might represent a distinct species. Although Ohdachi et al. (2006) observed phylogenetic differentiation between the two samples they used from Taiwan and Vietnam, these authors continued to consider the Taiwanese samples as a subspecies of *Crocidura attenuata*.

Esselstyn and Brown (2009) and Esselstyn et al. (2009) studying Southeast Asian shrews, recognised the relationship between samples of *Crocidura tanakae* from Taiwan and a sample from northeastern Vietnam, which they identified in these studies as *Crocidura* cf. *tanakae*. The following year, Esselstyn and Oliveros (2010) demonstrated the presence of two similar sized species of *Crocidura* in Vietnam, namely *Crocidura attenuata* based on samples from northern Vietnam and *Crocidura* cf. *tanakae* based on samples from four separate localities in northern and central Vietnam. Bannikova et al. (2011) included their own recently collected samples from northern, central and southern Vietnam plus information derived from GenBank. They were similarly able to demonstrate the presence of two separate species, *Crocidura attenuata* confined to a single locality in northeastern Vietnam and *Crocidura tanakae* which was widespread in northern, central and southern localities. With a minimum distance of 9.91% between the haplotypes, their cyt *b* tree showed good support for the distinction of *Crocidura tanakae* from a multi-species group comprising *Crocidura attenuata*, *Crocidura dsinezumi*, *Crocidura indochinensis*, *Crocidura lasiura*, *Crocidura tadae kuroda*, *Crocidura wuchihensis*, *Crocidura* sp. AB1 and *Crocidura zaitsevi*.

### *Crocidura wuchihensis*, *Crocidura indochinensis* and *Crocidura* sp. AB1

Although known from few specimens at any one location *Crocidura indochinensis* Robinson & Kloss, 1922 was considered to have a wide, disjunct distribution, occuring in a few widely separated locations in Vietnam and extralimitally in Myanmar and China (Osgood 1932, Anthony 1941, Heaney and Timm 1983, Jiang and Hoffmann 2001, Hutterer 2005, Jenkins et al. 2009). Additional specimens from southern Vietnam were recorded recently (Abramov et al. 2009; Jenkins et al. 2010) and tissue samples from these specimens were included in the molecular analysis by Bannikova et al. (2011).

*Crocidura wuchihensis* Shaw, Wang, Lu and Chang, 1966 was originally described on the basis of two specimens from Hainan Island, China. Specimens collected from two localities in Vietnam (Lunde et al. 2003; 2004) were referred to this species and specimens from several other locations in Vietnam were also considered to represent *Crocidura wuchihensis* (Jenkins et al. 2009). Samples of *Crocidura wuchihensis* from Ha Giang Province, Mt. Tay Con Linh II, northern Vietnam were included in molecular analyses of Ohdachi et al. (2006) and Esselstyn and Oliveros (2010). A separate analysis of a sample from Vinh Phu Province, Tam Dao, northern Vietnam (Meegaskumbura et al. 2007) from a specimen misidentified as *Crocidura fuliginosa*, also proved to be a representative of *Crocidura wuchihensis* (see Bannikova et al. 2011). Bannikova et al. (2011) added GenBank data from samples from these two localities to their cyt *b* analysis of samples from northern, central and southern Vietnam. Their analysis of cyt *b* revealed the presence of three taxa forming a group with 100% support: *Crocidura wuchihensis* in the two localities (Ha Giang Province and Vinh Phu Province) in northern Vietnam; *Crocidura indochinensis* in a single locality (Bi Doup - Nui Ba Nature Reserve) in southern Vietnam; and an unnamed species, designated as *Crocidura* sp. AB1, from Sa Pa in northern Vietnam. The average distance on the cyt *b* tree between *Crocidura wuchihensis* and the combined *Crocidura indochinensis*/*Crocidura* sp. AB1 branch was 7.75%. The *p-* distance from the cyt *b* tree separating *Crocidura wuchihensis* and *Crocidura indochinensis* is 7.6%, and separating *Crocidura wuchihensis* and *Crocidura* sp. AB1 was 8.0%. There was 100% bootstrap support on the cyt *b* and COI trees for the *Crocidura indochinensis* and *Crocidura* sp. AB1 group but these two taxa were respectively separated at *p*-distances of 4.1% (for cyt *b*) and 4.0% (in the COI analysis).

## Methods

**Figure 1.**
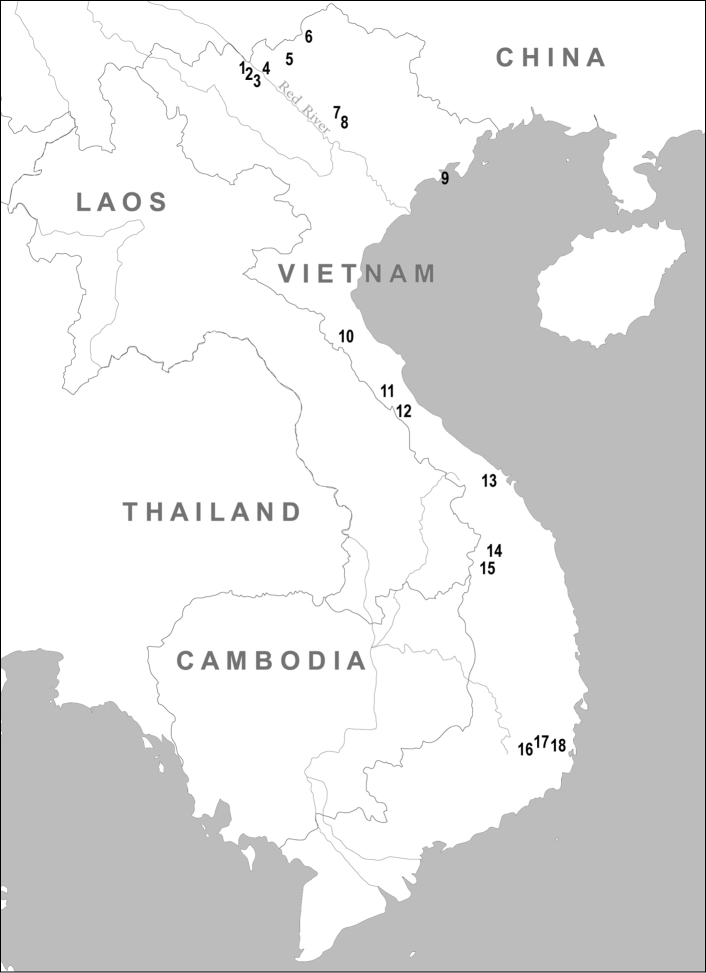
Geographical distribution of sampling localities in Vietnam: **1** Lao Cai Province, Ngai Tio **2** Lao Cai Province, Sa Pa District **3** Lao Cai Province, Van Ban District **4** Lao Cai Province, Thai Nien **5** Lao Cai Province, Pa Kha **6** Ha Giang Province, Mt. Tay Con Linh II **7** Tuyen Quang Province **8** Vinh Phu Province, Tam Dao **9** Hai Phong Province, Cat Ba Island **10** Ha Tinh Province, Huong Son District **11** Quang Binh Province, Phong Nha - Ke Bang National Park **12** Quang Tri Province, Huong Hoa Nature Reserve **13** Quang Nam – Da Nang Provinces, Ba Na Nature Reserve **14** Kon Tum Province, Ngoc Linh Mt. **15** Kon Tum Province, Dak To **16** Lam Dong Province, Da Lat **17** Lam Dong Province, Bi Doup - Nui Ba Nature Reserve **18** Khanh Hoa Province, Hon Ba Mt.

This morphological study draws on specimens from a wide range of geographical locations in Vietnam (see Fig. 1) and includes those specimens from which tissue samples were analysed in the papers by Ohdachi et al. (2006), Meegaskumbura et al. (2007), Esselstyn et al. (2009), Esselstyn and Brown (2009), Esselstyn and Oliveros (2010) and Bannikova et al. (2011). The specimens included in this study (see supplementary file) are stored in the collections of the American Museum of Natural History, New York (AMNH); Natural History Museum, London (BMNH); Field Museum of Natural History, Chicago (FMNH); Museum of Vertebrate Zoology, University of California, Berkely (MVZ); National Museum of Natural History, Smithsonian Institution, Washington D.C. (USNM); Zoological Institute, Russian Academy of Sciences, Saint-Petersburg, Russia (ZIN).

Measurements in millimetres were taken with digital callipers. Cranial and dental nomenclature follows that of Meester (1963), Mills (1966), Butler and Greenwood (1979) and Dannelid (1998). Definitions of skull measurements follow Jenkins et al. (2009).

## Results

### Crocidura sapaensis sp. n.

urn:lsid:zoobank.org:act:6016D7F3-D50E-4DBC-B571-EB54E5062D1E

http://species-id.net/wiki/Crocidura_sapaensis

#### Holotype.

ZIN 96433, genetic analysis code CVN108, BOLD Accession no. ABMIV114-08, field no. 132, male, body in ethanol, skull extracted, collected 25 May 2006 by A.V. Abramov.

#### Type locality.

Vicinity of Tram Ton Station of Hoang Lien National Park, north slope of Phansipan Mt. area, 6 km west of Sa Pa Town, Sa Pa District, Lao Cai Province, Vietnam, 22°21'N, 103°46'E, altitude 2200m above sea level.

#### Paratypes.

ZIN 96262, genetic analysis code CVN93, GenBank no. HM587005, BOLD no. ABMIV100-08, field no. 13, male, collected 8 December 2005; ZIN 96264, genetic analysis code CVN94, GenBank no. HM587006, BOLD no. ABMIV101-08, field no. 15, female, collected 8 December 2005; ZIN 96269, genetic analysis code CVN99, BOLD no. ABMIV106-08, field no. 32, male, collected 15 December 2005; ZIN 96271, genetic analysis code CVN101, BOLD no. ABMIV108-08, female, collected 16 December 2005; ZIN 96274, genetic analysis code CVN102, BOLD no. ABMIV109-08, field no. 45, male, collected 17 December 2005; ZIN 96275, genetic analysis code CVN103, BOLD no. ABMIV110-08, field no. 46, male, collected 17 December 2005; ZIN 96276, genetic analysis code CVN104, BOLD no. ABMIV111-08, field no. 66, female, collected 22 December 2005; ZIN 96432, genetic analysis code CVN107, BOLD no. ABIOW074-08, field no. 131, male, collected 25 May 2006; ZIN 96434, genetic analysis code CVN109, BOLD no. ABIOW075-08, field no. 133, male, collected 25 May 2006; ZIN 96436, genetic analysis code CVN111, BOLD no. ABMIV116-08, field no. 136, male, collected 28 May 2006; ZIN 96438, genetic analysis code CVN113, BOLD no. ABMIV117-08, field no. 138, female, collected 28 May 2006; ZIN 96439, genetic analysis code CVN114, BOLD no. ABMIV118-08, field no. 139, female, collected 28 May 2006; ZIN 96442, genetic analysis code CVN117, BOLD no. ABIOW069-08, field no. 144, male, collected 31 May 2006; ZIN 99779, field no. 24, male, collected 10 May 2010. All bodies in ethanol, skulls extracted, collected by A.V. Abramov and A.V. Shchinov from the same locality as the holotype, altitude 1930–2200m above sea level.

**Other material.** FMNH 39029 Chapa [Sa Pa], Lao Cai Province; BMNH 1925.1.1.24; BMNH 1925.1.1.27 Ngai Tio, Lao Cai Province, 22°36'N, 103°40'E.

#### Diagnosis.

A small shrew distinguished by the mitochondrial genes cytochrome *b* (cyt *b*) and cytochrome oxidase *c* subunit I (COI) and by the shape of the talonid of the third lower molar (m3).

#### Description.

**Figure 2.**
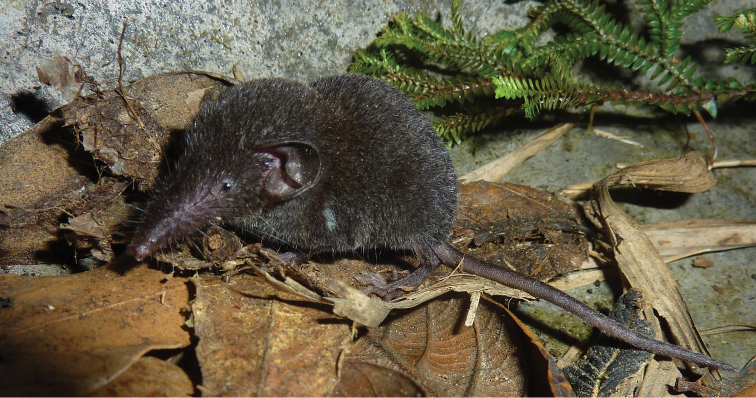
Photograph of adult male *Crocidura sapaensis* (ZIN 99779).

**Figure 3.**
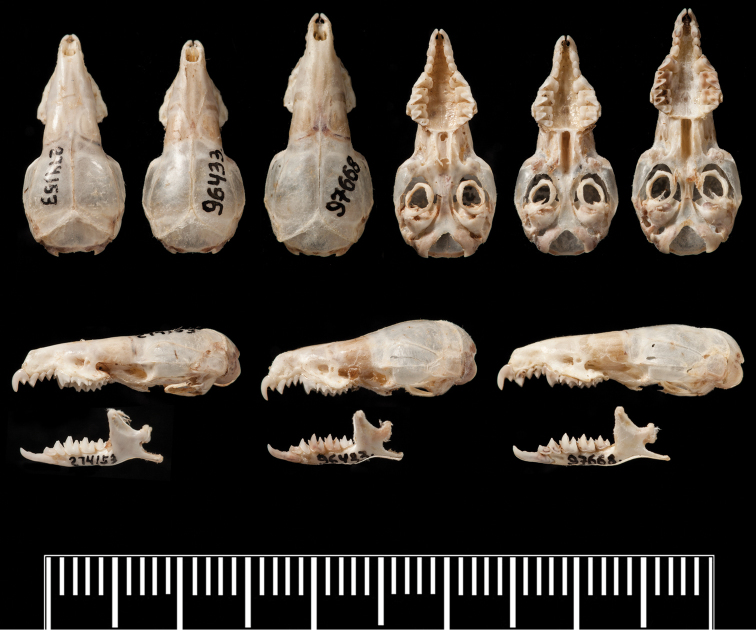
Comparison of crania of *Crocidura wuchihensis* (AMNH 274153), *Crocidura sapaensis* (ZIN 96433) and *Crocidura indochinensis* (ZIN 97668). Top row from left to right: dorsal views of the skulls of *Crocidura wuchihensis*, *Crocidura sapaensis* and *Crocidura indochinensis*, ventral views of the skulls in the same order. Lower row: left lateral view of skulls and mandibles from left to right of *Crocidura wuchihensis*, *Crocidura sapaensis* and *Crocidura indochinensis*.

**Figure 4.**
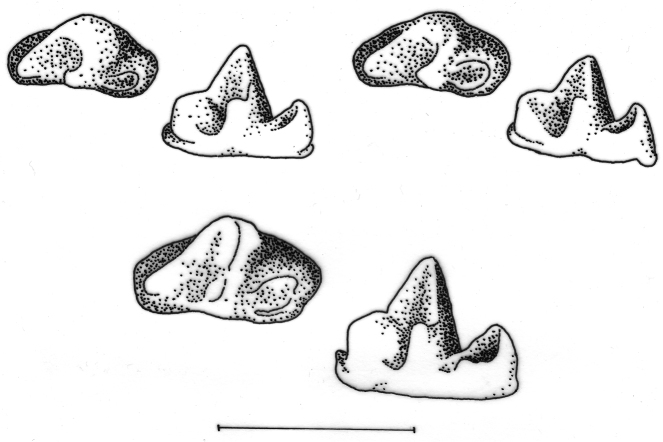
Occlusal (left) and lingual (right) views of right lower third molar to show differences in development of the talonid. Upper row left *Crocidura wuchihensis* AMNH 274168; upper row right *Crocidura sapaensis* ZIN 96439; lower row *Crocidura indochinensis* ZIN 97671. Scale equals 1 mm.

Size small (see Table 1) with a moderately long tail relative to head and body length (62–84%). Dorsal pelage dark greyish brown; tail dark grey dorsally, slightly paler below (see Fig. 2). Skull with a rounded, short rostrum: moderately broad interorbital region; rounded, relatively deep braincase with subangular superior articular facets and lambdoid crests just evident laterally near the junction with the mastoid (see Fig. 3). The first upper incisor is slender with a relatively small posterior cusp, less than half the height of the first upper unicuspid; posterolingual border of upper premolar (P4) deep and rounded, in close contact with the anterolingual margin of M1 in occlusal view; last upper molar (M3) relatively narrow. Lower incisor with two distinct cusps on the occlusal surface in unworn dentition; posterolingual cuspid present on lower premolar (p4); talonid basin of m3 broad and deep with an entoconid ridge and low entoconid (see Fig. 4).

**Table 1.** Comparison of *Crocidura indochinensis*, *Crocidura wuchihensis* and *Crocidura sapaensis*. *Crocidura guy* and *Crocidura zaitsevi* B are included as representatives of the very small species in Vietnam. Measurements in millimetres are presented as the mean, standard deviation and range, followed by sample size in parentheses.<br/>

**Character**	***Crocidura zaitsevi* B Bi Doup & Hon Ba**	***Crocidura guy* Na Hang**	***Crocidura wuchihensis* Mt Tay Con Linh II**	***Crocidura sapaensis* Sa Pa**	***Crocidura indochinensis* Bi Doup**
Condyloincisive length	15.4 ± 0.28<br/> 14.9-15.8 (15)	15.4 ± 0.05<br/> 15.3-15.4 (4)	16.4 ± 0.5<br/> 15.7-17.1 (6)	16.6 ± 0.41<br/> 15.6-17.2 (20)	18.1 ± 0.36<br/> 17.5-18.7 (10)
Condylobasal length	14.8 ± 0.31<br/> 14.2-15.3 (15)	14.9 ± 0.06<br/> 14.8-14.9 (4)	15.7 ± 0.47<br/> 15.0-16.4 (6)	15.9 ± 0.42<br/> 15.0-16.5 (20)	17.4 ± 0.37<br/> 16.8-17.8 (10)
Upper toothrow length	6.5 ± 0.14<br/> 6.3-6.8 (15)	6.5 ± 0.14<br/> 6.4-6.7 (4)	7.0 ± 0.25<br/> 6.6-7.2 (6)	7.0 ± 0.17<br/> 6.5-7.2 (21)	7.7 ± 0.23<br/> 7.3-8.0 (10)
Maxillary breadth at M2	4.5 ± 0.19<br/> 4.2-4.9 (15)	4.5 ± 0.14<br/> 4.4-4.7 (4)	4.9 ± 0.06<br/> 4.8-5 (6)	4.8 ± 0.18<br/> 4.4-5.1 (21)	5.2 ± 0.07<br/> 5.1-5.3 (10)
Braincase breadth	7.2 ± 0.15<br/> 6.9-7.5 (15)	7.2 ± 0.17<br/> 7.0-7.4 (4)	7.5 ± 0.16<br/> 7.3-7.8 (6)	7.7 ± 0.19<br/> 7.4-8.1 (19)	8.2 ± 0.15<br/> 7.9-8.4 (10)
Braincase height	3.7 ± 0.13<br/> 3.5-3.9 (14)	3.6 ± 0.14<br/> 3.5-3.8 (4)	4.0 ± 0.15<br/> 3.7-4.1 (6)	4.1 ± 0.17<br/> 3.9-4.4 (19)	4.4 ± 0.14<br/> 4.1-4.5 (10)
Head and body length	52.7 ± 2.79<br/> 49-59 (15)	49.5 ± 2.27<br/> 47-53 (4)	60.6 ± 2.7<br/> 58-65 (5)	57.4 ± 3.91<br/> 50-65 (20)	63.5 ± 3.81<br/> 56-68 (10)
Tail length	31.8 ± 1.2<br/> 30-34 (15)	35.9 ± 1.4<br/> 34-37 (4)	39.8 ± 2.28<br/> 37-42 (5)	41.6 ± 2.48<br/> 37-47 (20)	55 ± 2.79<br/> 50-58 (10)
Ratio of tail length to head and body length	0.61 ± 0.02<br/> 0.55-0.65 (15)	0.73 ± 0.03<br/> 0.69-0.77 (4)	0.66 ± 0.04<br/> 0.63-0.72 (5)	0.73 ± 0.06<br/> 0.62-0.84 (20)	0.87 ± 0.05<br/> 0.81-0.98 (10)
Ratio of tail length to condyloincisive length	2.1 ± 0.06<br/> 2.0-2.2 (15)	2.3 ± 0.09<br/> 2.2-2.4 (4)	2.4 ± 0.14<br/> 2.2-2.6 (5)	2.5 ± 0.14<br/> 2.2-2.7 (20)	3.1 ± 0.12<br/> 2.9-3.2 (10)

#### Comparison with other species.

**Figure 5.**
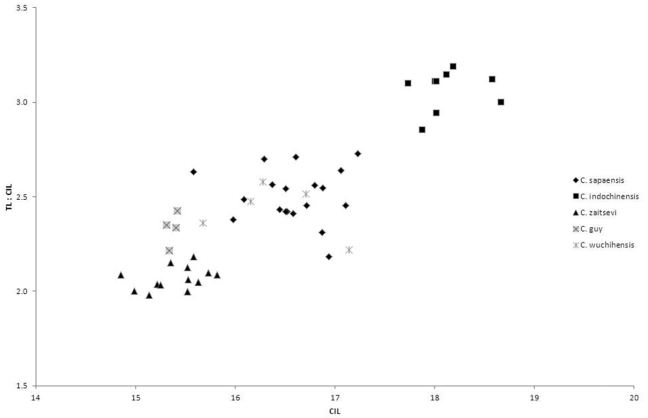
Bivariate plot to show differences in skull size and relative tail length. Horizontal axis: condyloincisive length; vertical axis: ratio of tail length to condyloincisive length.

*Crocidura sapaensis* averages larger than the very small species of *Crocidura* recorded from Vietnam. The condyloincisive length is greater than that of *Crocidura guy*, *Crocidura annamitensis* and *Crocidura kegoensis*, within the upper part of the range of *Crocidura zaitsevi* and the braincase is deeper than that of all four small species. *Crocidura wuchihensis* and *Crocidura sapaensis* are in the same size range. *Crocidura sapaensis* is smaller than or at the lower end of the size range of *Crocidura indochinensis* with a relatively shorter tail (see Table 1 and Fig. 5).

*Crocidura sapaensis* and *Crocidura wuchihensis* are distinguished by differences in cyt *b* sequences. *Crocidura sapaensis* differs from *Crocidura zaitsevi* and *Crocidura indochinensis* in the cyt *b* and COI gene sequences.

Differences in the shape of the talonid of m3 in northern Vietnamese populations serve to distinguish *Crocidura sapaensis* and *Crocidura wuchihensis* (see Fig. 4). In specimens of *Crocidura sapaensis* from northern Vietnam the talonid basin is broad and deep with an entoconid ridge and low entoconid, whereas in *Crocidura wuchihensis* the talonid basin is narrow. In *Crocidura indochinensis* the talonid basin is broad and deep with a hypoconid, entoconid and marked entoconid ridge (see Fig. 4).

#### Etymology.

The new species is named after Sa Pa, the capital of Sa Pa District in Lao Cai Province of northern Vietnam, with the Latin suffix - *ensis* (belonging to).

#### Natural history.

**Figure 6.**
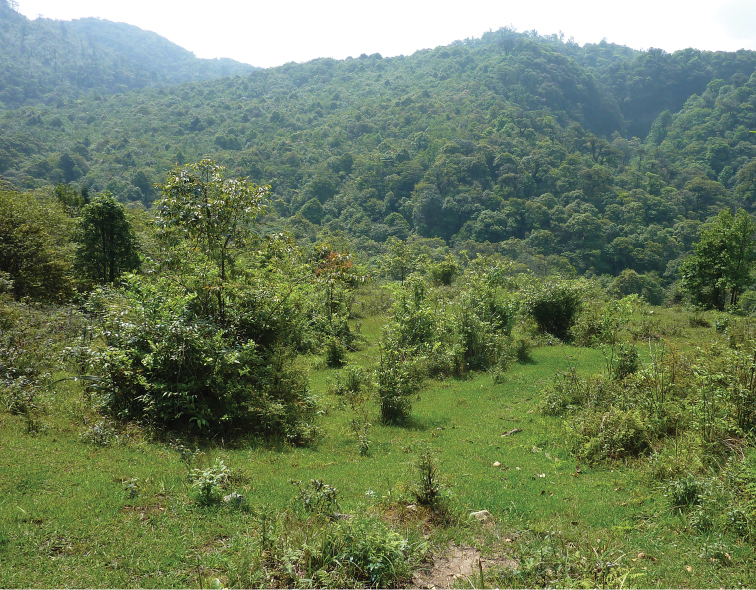
Habitat typical of the area where *Crocidura sapaensis* was found.

The series of type specimens was collected from a variety of habitats in the vicinity of Tram Ton Station of Hoang Lien National Park: mixed evergreen forest; forested banks of small streams; open grassy glades (Fig. 6); primary forest with large trees at an elevation 1930–2200m (Abramov et al. 2008b). During 2005–2010 a total of 190 shrews was captured in this area, including 4 species (*Crocidura sapaensis*, *Blarinella griselda* Thomas, 1912, *Anourosorex squamipes* Milne Edwards, 1872, and *Episoriculus leucops* (Horsfield 1855)). *Crocidura sapaensis* was the most numerous species (90% of the total captures), followed by *Anourosorex squamipes* and *Blarinella griselda* (5.3% and 4.2% respectively), while only one *Episoriculus leucops* was captured (Abramov et al. 2010). *Crocidura sapaensis* was more abundant in slightly disturbed mixed forest (2.2–3.0 specimens per 100 trap/nights), the occurrence in open glades, amongst shrubs on stream banks and in undisturbed primary forest was 0.3–2.6 specimens per 100 trap/nights. The proportion of males to females in *Crocidura sapaensis* was greater in all seasons; on average the male to female ratio is 2.3. Pregnant females were recorded from May to mid-July. Mean litter size in *Crocidura sapaensis* was 3.0 (2-4, n=15).

#### Distribution.

Confirmed specimens of *Crocidura sapaensis* are recorded from Lao Cai Province, Sa Pa District on the basis of cyt *b* analysis and morphology of m3. On the basis of morphology, specimens from the northern part of Lao Cai Province, Ngai Tio (elevation 1450m) and from the vicinity of Cat Cat Village near Sa Pa Town (elevation 1400–1450m) in relatively close geographical proximity also probably belong to the same species.

Populations of *Crocidura wuchihensis* identified on the basis of cyt *b* and those probably representing this species on the basis of morphology (from Pa Kha and Thai Nien, both in Lao Cai Province) all occur in northeastern Vietnam in localities to the east of the Song Hong (Red River). The observation that this river marks the border between the two species, with *Crocidura wuchihensis* to the east and *Crocidura sapaensis* to the west, was made by Bannikova et al. (2011), however this apparent biogeographical separation is based on few locality records. These authors also observed that, in the cyt *b* analysis, the two northern Vietnamese populations of *Crocidura wuchihensis* (from Mt Tay Con Linh II [22°46'N, 104°49'E] and Tam Dao [21°27'N, 105°38'E]) were separated by a *p-* distance of 2.1% suggesting that they probably represent distinct geographic populations.

The population of *Crocidura wuchihensis* recorded from Huong Son, Ha Tinh Province in the southern Annamites by Lunde et al. (2004) and Jenkins et al. (2009), does of course, occur west of the Song Hong and samples have not been included in any of the previous molecular studies. Specimens from Mt Tay Con Linh II are larger on average (CIL 15.7–17.1, mean 16.4) than those from Huong Son (CIL 15.8-16.4, mean 16.0). The Canonical Variate Analysis reported in Jenkins et al. (2009: Fig. 10) shows that these two groups respectively from northern Vietnam and the southern Annamites are moderately well separated from each other. In view of the problems outlined in this paper, lacking further evidence from molecular studies, it is impossible to predict if the population from Huong Son is correctly assigned to *Crocidura wuchihensis*, could belong to *Crocidura sapaensis*, or might indeed represent a further undescribed species.

##### *Crocidura attenuata* and *Crocidura tanakae* Characters separating *Crocidura attenuata* and *Crocidura tanakae*

**Figure 7.**
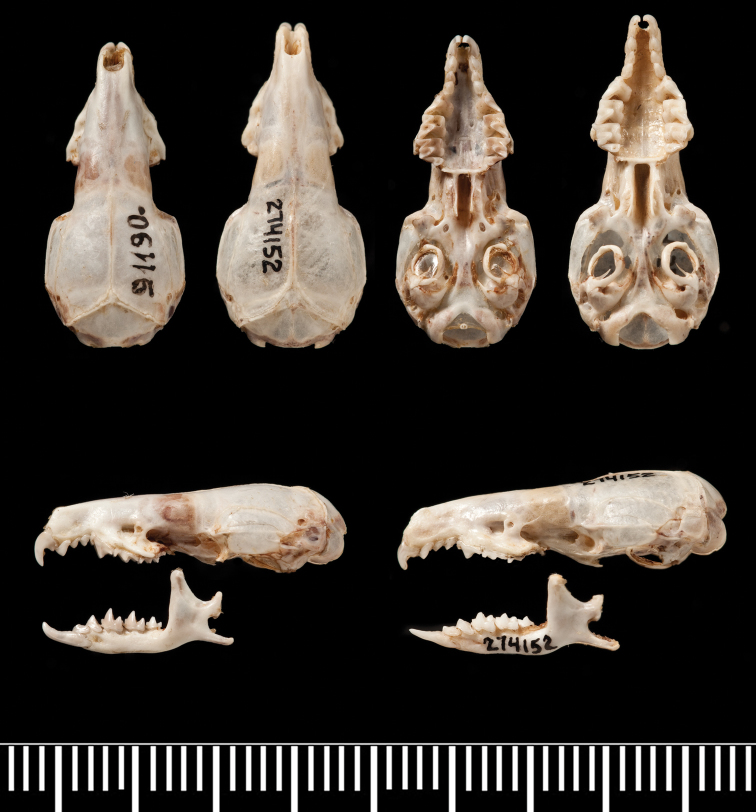
Comparison of crania of *Crocidura tanakae* (ZIN 91190) and *Crocidura attenuata* (AMNH 274152). Top row from left to right: dorsal views of the skulls of *Crocidura tanakae* and *Crocidura attenuata*, ventral views of the skulls in the same order. Lower row: left lateral view of skulls and mandibles from left to right of *Crocidura tanakae* and *Crocidura attenuata*.

**Figure 8.**
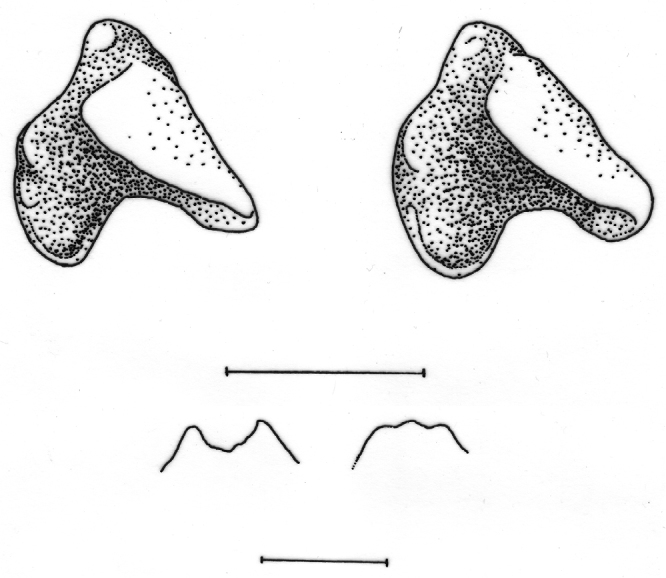
Above: occlusal view of left upper premolar of *Crocidura tanakae* (ZIN 91205) left and *Crocidura attenuata* (AMNH 274232) right. Below: palatal sutures of the same specimens in the same order. Scales equal 1 mm.

The population from Mt Tay Con Linh II, Ha Giang Province in northern Vietnam recognised by molecular analysis of cyt *b* as *Crocidura attenuata*, falls within the size range of *Crocidura tanakae* and both species are morphologically very similar in appearance. The two species may be separated by the following characters. The basioccipital region in *Crocidura attenuata* is narrow and ridged particularly anterior to the position of the basioccipital suture, whereas in *Crocidura tanakae* the basioccipital region is broad and flat to concave (see Fig. 7). The palatal suture in *Crocidura attenuata* is a rounded to flat-topped ‘n’ shape, whereas in *Crocidura tanakae* the suture is a shallow to more marked ‘m’ shape (see Fig. 8). The two species also differ in the shape of the talon of the upper premolar (P4). In occlusal view, the talon of *Crocidura attenuata* is broader and more angular than that of *Crocidura tanakae*, the lingual border is straight to concave, with the posterior border shallowly indented so that the whole tooth looks larger in occlusal view. In *Crocidura tanakae* the lingual border of P4 is rounded and the posterior border of the tooth is deeply indented (see Fig. 8).

##### Geographical variation in *Crocidura tanakae*

Esselstyn and Oliveros (2010) did not provide detailed results about Vietnamese samples in the text of their analysis of Asian *Crocidura tanakae*, nevertheless they demonstrated apparent geographical variation in Vietnam. Their illustration of a statistical parsimony network of mitochondrial haplotypes (Esselstyn and Oliveros 2010: Fig. 4) shows northeastern Vietnam samples from Tam Dao (21°27'N, 105°38'E) and Tuyen Quang (22°20'N, 105°25'E) grouped relatively closely, separated from each other by relatively few steps but separated by multiple steps from samples from the other two localities, Ha Tinh (18°21'N, 105°13'E) and Quang Nam (15°12'N, 108°02'E), which form a looser group. In their analysis of the COI gene, Bannikova et al. (2011) demonstrated the presence of two clearly defined haplogroups within Vietnamese *Crocidura tanakae*: *Crocidura tanakae* B restricted to the northern part of the country (Hoang Lien Mountains, Van Ban District) and *Crocidura tanakae* A which was more widespread in Central and South Vietnam (Huong Hoa, Phong Nha-Ke Bang, Ngoc Linh, Hon Ba and Bi Doup). The uncorrected *p*-distance between these two groups using the COI gene was about 2.5%.

Populations of *Crocidura tanakae* in Vietnam show a distinct clinal variation in skull size, populations at higher latitudes averaging smaller in size than those at lower latitudes (see Table 2). Although sample sizes are small, this observation is a possible example of the converse Bergmann’s rule where body size decreases with latitude.

**Table 2.** Latitudinal size variation in *Crocidura tanakae*. Measurements in millimetres are presented as the mean, standard deviation and range, followed by sample size in parentheses.<br/>

**Character**	**Lao Cai Prov., Van Ban Dist. 21°58'N**	**Ha Tinh Prov., Huong Son 18°21'N**	**Quang Tri Prov., Huong Hoa 16°56'N**	**Kon Tum Prov., Ngoc Linh 15°05'N**	**Lam Dong Prov,. Bi Doup 12°11'N**
Condyloincisive length	19.3 ± 0.47<br/> 18.5–20.0 (14)	19.7 ± 0.47<br/> 18.4–20.4 (24)	20.1 ± 0.38<br/> 19.6–20.6 (5)	20.6 ± 0.36<br/> 20.1–20.9 (4)	20.4 ± 0.52<br/> 19.6–21.3 (11)
Condylobasal length	18.8 ± 0.48<br/> 17.9–19.4 (14)	18.9 ± 0.48<br/> 17.7–19.6 (24)	19.2 ± 0.36<br/> 18.8–19.6 (5)	19.8 ± 0.29<br/> 19.4–20.0 (4)	19.5 ± 0.52<br/> 18.8–20.6 (11)
Upper toothrow length	8.3 ± 0.18<br/> 7.9–8.6 (14)	8.5 ± 0.22<br/> 8.2–9.0 (24)	9.0 ± 0.24<br/> 8.6–9.3 (5)	9.1 ± 0.14<br/> 8.9–9.2 (4)	9.0 ± 0.23<br/> 8.5–9.4 (11)
Maxillary breadth at M2	5.9 ± 0.17<br/> 5.6–6.3 (14)	6.0 ± 0.19<br/> 5.6–6.4 (24)	6.2 ± 0.17<br/> 6.0–6.4 (5)	6.3 ± 0.18<br/> 6.1–6.5 (4)	6.2 ± 0.21<br/> 5.9–6.6 (11)
Braincase breadth	8.7 ± 0.31<br/> 8.3–9.2 (14)	8.9 ± 0.19<br/> 8.3–9.1 (24)	9.0 ± 0.14<br/> 8.7–9.1 (5)	9.2 ± 0.36<br/> 8.9–9.6 (4)	9.3 ± 0.26<br/> 8.9–9.6 (11)

##### Geographical variation in *Crocidura attenuata*

Abramov et al. (2012) demonstrated apparent geographical variation of *Crocidura attenuata* in southern China and northern Vietnam. Genetic differentiation of *Crocidura attenuata* is notable and reveals a phylogeographic structure with four haplogroups. The specimens from Cat Ba Island (Hai Phong Province, northeastern Vietnam) formed a single cluster closely related to the group of specimens from northern Vietnam (Ha Giang Province) and southeastern China (Guangxi Province). The genetic distance (*p*-distance) between specimens from Cat Ba / Ha Giang as well as Cat Ba / Guangxi is about 2.1%. The specimen of *Crocidura attenuata* from the more north-eastern region of China (Hunan Province) appears basal among all samples of *Crocidura attenuata* from China and Vietnam. Thus, the genetic distance between two specimens from China (Hunan / Guangxi) is 4.3%, which is nearly the same as the distance between *Crocidura indochinensis* and *Crocidura sapaensis* (see Bannikova et al. 2011).

##### Distribution

While *Crocidura attenuata* in Vietnam appears to occur only to the east of the Song Hong (Red River), in northeastern Vietnam (Bannikova et al. 2011, Abramov et al. 2012), *Crocidura tanakae* does not appear to be so constrained and has been recorded on both sides of the river in northern Vietnam and also in central and southern Vietnam (Esselstyn and Oliveros 2010, Bannikova et al. 2011, this study).

## Discussion

For sister species that are recognised on the basis of morphology, cyt *b* distance values typically exceed 5% (Baker and Bradley 2006). The *p-* distance values of at least 9.91% between haplotypes belonging to the *Crocidura tanakae* and *Crocidura attenuata* groups, of 7.6% separating *Crocidura wuchihensis* and *Crocidura indochinensis*, and of 8.0% separating *Crocidura wuchihensis* and *Crocidura sapaensis* provide compelling evidence in support of their taxonomic distinction (Bannikova et al. 2011). The lower *p-* distance values of 4.1% separating *Crocidura indochinensis* from *Crocidura sapaensis* are somewhat less convincing, were it not for the ready morphological distinction of the two species. The conundrum is the morphological distinction yet relatively low molecular separation between *Crocidura indochinensis* and *Crocidura sapaensis*, in contrast to *Crocidura wuchihensis* and *Crocidura sapaensis* which are not easily defined by morphological features but are distinguished by high molecular values. Similar situations are found in the literature, for example amongst different species of *Eumops* Miller, 1906 (bonneted bats) (McDonough et al. 2008). It is possible to speculate that this implies a relatively closer relationship between *Crocidura indochinensis* and *Crocidura sapaensis* but morphological convergence of *Crocidura wuchihensis* and *Crocidura sapaensis* to meet similar ecological requirements.

*Crocidura tanakae* is currently recognised as being widely distributed in Southeast Asia including southern China, Vietnam, Taiwan and the Philippines (Esselstyn and Oliveros 2010, Bannikova et al. 2011). *Crocidura attenuata* has a more northerly distribution (Esselstyn and Oliveros 2010) and furthermore evidence suggests that, at least in Vietnam, the distribution may be more restricted than formerly understood (Bannikova et al. 2011, this study). It is conceivable that these two morphologically convergent species have secondarily come into contact with each other as there is evidence that the two species are sympatric in at least one Chinese locality (Esselstyn and Oliveros 2010, Judith Eger pers. comm.).

## Supplementary Material

XML Treatment for Crocidura sapaensis

## Appendix

Specimens included in the morphological study. (doi: 10.3897/zookeys.313.4823.app). File format: Microsoft Word document (doc). **Explanation note:** The supplementary file contains a list of all specimens included in this study.
