# Supplementary material for: Bones and genes: resolution problems in three Vietnamese species of Crocidura (Mammalia, Soricomorpha, Soricidae) and the description of an additional new species
Source: Zookeys. 2013 Jul 2;(313):61–79. doi: 10.3897/zookeys.313.4823 (PMC3701231; doi:10.3897/zookeys.313.4823)
Supplement: Supplementary file 2 — Specimens included in the morphological study. (doi: 10.3897/zookeys.313.4823.app). File format: Microsoft Word document (doc). [file ZooKeys-313-061-s001.doc]

Supplementary file

Specimens included in the morphological study. Tissues of specimens marked * were included in molecular analyses.

Acronyms prefacing specimen numbers:

AMNH - American Museum of Natural History, New York, USA

AMCC - Ambrose Monell Cryo Collection, American Museum of Natural History, New York, USA

BMNH - The Natural History Museum, London, UK

FMNH - Field Museum of Natural History, Chicago, USA

MVZ - Museum of Vertebrate Zoology, University of California, Berkeley, USA

ZIN - Zoological Institute, Russian Academy of Sciences, Saint-Petersburg, Russia

*Crocidura attenuata*

Ha Giang Province, Mt. Tay Con Linh II (22° 45' 47" N, 104° 49' 49" E): *AMNH 274146, AMCC 101492; *AMNH 274147, AMCC 101493 (Ohdachi *et al*., 2006; Esselstyn and Oliveros, 2010; Bannikova *et al*., 2011); AMNH 274148; AMNH 274151; AMNH 274152; AMNH 274229; AMNH 274232; AMNH 274233; AMNH 274345; AMNH 274346; AMNH 274347; AMNH 274348; AMNH 274249.

*Crocidura indochinensis*

Lam Dong Province, Da Lat (11° 56' N, 108° 25' E): BMNH 1947.1424.

Lam Dong Province, Lac Duong District, Bi Doup - Nui Ba Nature Reserve (12º 11' N, 108º 41' E): ZIN 97667; *ZIN 97668; *ZIN 97669; *ZIN 97670; *ZIN 97671; *ZIN 97672; *ZIN 97673; *ZIN 97674; *ZIN 98962 (Bannikova *et al*., 2011).

*Crocidura sapaensis*

Lao Cai Province, Sa Pa District (22º 21' N, 103º 46' E): FMNH 39029; *ZIN 96262; *ZIN 96264; ZIN 96266; ZIN 96267; *ZIN 96269; *ZIN 96271; *ZIN 96274; *ZIN 96275; *ZIN 96276; *ZIN 96432; *ZIN 96433; *ZIN 96434; ZIN 96435; *ZIN 96436; ZIN 96437; *ZIN 96438; *ZIN 96439; *ZIN 96442; ZIN 96443; ZIN 96498 (Bannikova *et al*., 2011).

Lao Cai Province, Ngai Tio (22° 36' N, 103° 40' E): BMNH 1925.1.1.24; BMNH 1925.1.1.27.

*Crocidura tanakae*

Lao Cai Province, Van Ban District, Nam Xay Commune (21º 58' N, 104º 02' E): *ZIN 91190; *ZIN 91193; *ZIN 91194; *ZIN 91195; *ZIN 91198; *ZIN 91201; *ZIN 91202; *ZIN 91204; *ZIN 91205; *ZIN 91206; *ZIN 91207; *ZIN 91208; *ZIN 91209; *ZIN 91210 (Bannikova *et al*., 2011).

Vinh Phu Province, Vinh Yen District, Tam Dao (21º 27' N, 105º 38' E): *MVZ 185237 (Meegaskumbura *et al*., 2007; Esselstyn *et al*., 2009; Esselstyn and Brown, 2009; Esselstyn and Oliveros, 2010; Bannikova *et al*., 2011).

Ha Tinh Province, Huong Son District, Huong Son Camp (18° 21' 53" N, 105° 13' 13" E): AMNH 272121; AMNH 272123; AMNH 272124; AMNH 272126; AMNH 272132; AMNH 272141; AMNH 272150; AMNH 272172; AMNH 272203; AMNH 272223; AMNH 272320; AMNH 272371; AMNH 272372; *AMNH 272433, AMCC 110774 (Esselstyn and Oliveros, 2010); *AMNH 272434, AMCC 110775 (Esselstyn and Oliveros, 2010); AMNH 272513; AMNH 272516; AMNH 272517; AMNH 272568; AMNH 272585; AMNH 272599; AMNH 272600; AMNH 272613; AMNH 272646.

Quang Binh Province, Phong Nha - Ke Bang National Park (17º 38' N, 106º 06' E): *ZIN 97510 (Bannikova *et al*., 2011).

Quang Tri Province, Huong Hoa Nature Reserve (16º 56' N, 106º 35' E): *ZIN 97505; *ZIN 97506; *ZIN 97507; *ZIN 97508 (Bannikova *et al*., 2011); ZIN 97509.

Quang Nam - Da Nang Provinces, Ba Na Nature Reserve (15° 57' - 16° 03' N, 107° 57' - 108° 03' E): BMNH 1997.639.

Kon Tum Province, Ngoc Linh Mt. (15º 05' N, 107º 57' E): *ZIN 91229; *ZIN 91230; *ZIN 96409; *ZIN 96411 (Bannikova *et al*., 2011).

Kon Tum Province, Dak To (14° 42' N, 107° 51' E): BMNH 1926.10.4.45.

Lam Dong Province, Lac Duong District, Bi Doup - Nui Ba National Park (12º 11' N, 108º 41' E - 12º 11' N, 108º 48' E): *ZIN 97608; *ZIN 97613; *ZIN 97614; *ZIN 97615; ZIN 97617; ZIN 97618; *ZIN 97619; *ZIN 97623; *ZIN 97624; ZIN 97626; *ZIN 97627 (Bannikova *et al*., 2011).

Khanh Hoa Province, Hon Ba Mt. (12º 07' N, 108º 57' E): *ZIN 96412 (Bannikova *et al*., 2011).

*Crocidura wuchihensis*

Ha Giang Province, Mt. Tay Con Linh II (22° 45' 47" N, 104° 49' 49" E): AMNH 273209; *AMNH 274153, AMCC 101499; *AMNH 274162 AMCC 101508 (Ohdachi *et al*., 2006; Esselstyn and Oliveros, 2010; Bannikova *et al*., 2011); AMNH 274167; AMNH 274168; AMNH 274182.

Lao Cai Province, Pa Kha (22° 33' N, 104° 16' E): BMNH 1933.4.1.168.

Lao Cai Province, Thai Nien (22° 24' N, 104° 05' E): BMNH 1925.1.1.29.

Vinh Phu Province, Vinh Yen District, Tam Dao (21º 27' N, 105º 38' E): *MVZ 186404 (Meegaskumbura *et al*., 2007; Bannikova *et al*., 2011).
